# JAK/STAT Pathway Targeting in Primary Sjögren Syndrome

**DOI:** 10.2478/rir-2022-0017

**Published:** 2022-10-20

**Authors:** Saviana Gandolfo, Francesco Ciccia

**Affiliations:** 1 Rheumatology Unit, Department of Internal Medicine, San Giovanni Bosco Hospital, Naples, Italy; 2 Department of Precision Medicine, Università della Campania Luigi Vanvitelli, Naples, Italy

**Keywords:** Sjögren's syndrome, JAK molecules, STAT molecules, cytokines

## Abstract

Primary Sjögren's syndrome (pSS) is an autoimmune systemic disease mainly affecting exocrine glands and resulting in disabling symptoms, as dry eye and dry mouth. Mechanisms underlying pSS pathogenesis are intricate, involving multiplanar and, at the same time, interlinked levels, e.g., genetic predisposition, epigenetic modifications and the dysregulation of both immune system and glandular-resident cellular pathways, mainly salivary gland epithelial cells. Unravelling the biological and molecular complexity of pSS is still a great challenge but much progress has been made in recent years in basic and translational research field, allowing the identification of potential novel targets for therapy development. Despite such promising novelties, however, none therapy has been specifically approved for pSS treatment until now. In recent years, growing evidence has supported the modulation of Janus kinases (JAK) - signal transducers and activators of transcription (STAT) pathways as treatment strategy immune mediated diseases. JAK-STAT pathway plays a crucial role in autoimmunity and systemic inflammation, being involved in signal pathways of many cytokines. This review aims to report the state-of-the-art about the role of JAK-STAT pathway in pSS, with particular focus on available research and clinical data regarding the use of JAK inhibitors in pSS.

## Introduction

Primary Sjögren's syndrome (pSS) is an autoimmune systemic disease which mainly affects exocrine glands and causes disabling symptoms, such as dry eye and dry mouth.^[1,2]^ Besides sicca, fatigue and pain are the other most common symptoms in pSS patients, together with a wide range of possible additional and highly heterogeneous clinical extra-glandular manifestations, potentially involving any organ.^[1,2]^ Furthermore, pSS patients are at higher risk than general population to develop lymphoma, mainly a non-Hodgkin B cell lymphoma of mucosa-associated lymphoid tissue (MALT) type.^[3,4]^ Females are most affected by pSS than males, with a ratio 9:1, but an increased risk of lymphoma development has been recently reported for the latter.^[5]^ The histological hallmark of pSS, i.e., the focal lymphocytic sialadenitis, observed in specimens obtained through minor salivary gland (MSG) biopsy, still represents, together with anti-SSA (Sjögren's-syndrome-related antigen A) autoantibodies, a crucial pillar for pSS classification.^[6]^

Mechanisms underlying pSS pathogenesis are intricate, involving multiplanar and, at the same time, interlinked levels, e.g., genetic predisposition, epigenetic modifications, and the dysregulation of both the immune system and glandular-resident cellular pathways, mainly salivary gland epithelial cells (SGECs).^[1,7,8,9]^ SGECs are not only damaged by the inflammatory process but are leading actors actively involved in several immune pathophysiology pSS processes.^[7,8]^ Infectious and/or exogenous agents might be involved in triggering the disease in predisposed individuals, enhanced by endogenous factors.^[10]^ Unraveling the biological and molecular complexity of pSS is still a great challenge, but much progress has been made in recent years in the basic and translational research field, allowing the identification of potential novel targets for therapy development. Despite such promising novelties,^[11]^ however, no therapy has been specifically approved for pSS treatment until now.^[12]^

In recent years, growing evidence has supported the modulation of Janus kinases (JAK)–signal transducers and activators of transcription (STAT) pathways as a treatment strategy for rheumatoid arthritis (RA) and spondyloarthritis (SpA), leading to the development of clinical trials and, finally, to the approval of different JAK inhibitors (JAK-i) agents for clinical use in RA and SpA.^[13,14,15]^ JAK-STAT pathway plays a crucial role in autoimmunity and systemic inflammation and are involved in signal pathways of many cytokines, and, for this reason, an increasing number of clinical trials with JAK-i have been performed even on other immune-mediated systemic diseases beyond RA and SpA, such as connective tissue diseases.^[16]^

This review aims to report the state of the art about the role of JAK-STAT pathway in pSS, with particular focus on available research and clinical data regarding the use of JAK-i in pSS.

## JAK-STAT Pathway and pSS Cytokine Landscape

The family of JAK-STAT molecules, including 4 JAK intracellular tyrosine kinases (i.e., JAK1, JAK2, JAK3, and TYK2) and seven transcription factors STAT (STAT1, STAT2, STAT3, STAT4, STAT5a and 5b, and STAT6), represents a crucial system involved in the transduction of signaling of several cytokines related to immune responses, inflammation, cell activation, and survival.^[17]^ Following the cytokine binding to its receptor on the cell membrane, JAK transfers phosphates from ATP (adenosine triphosphate) to intracellular domains of the cytokine receptor, to JAK members themselves, and to other downstream signaling molecules, such as STAT, that translocate to the nucleus and modulate the expression of defined gene sets.^[17]^

JAK-STAT dysregulation has been associated with different immune system disorders.^[18]^ Therefore, JAK-i, which causes competitive ATP binding and blocks the abovementioned cascade of events and gene expression has become an excellent novel therapeutic strategy in rheumatology.

**Figure 1:**
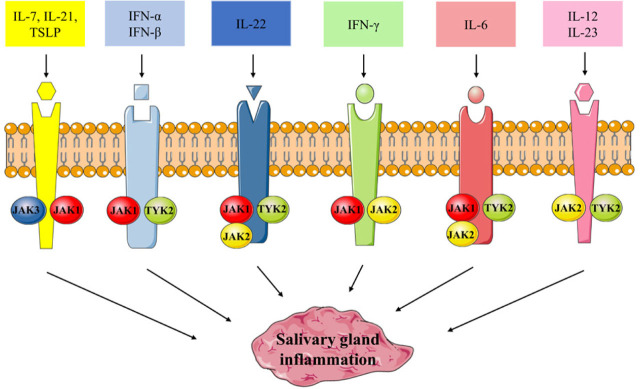
Main cytokines signaling through JAK-STAT involved in pSS pathogenesis.

Several JAK-i have been or are being developed, including first-generation pan-JAK-i, i.e., tofacitinb, baricitinib, ruxolitinib, and pefacitinib and second-generation more selective JAK-i, i.e., filgotinib, upadacitinib, and decernotinib. Tofacitinib acts by blocking JAK1 and JAK3 but has also a role in JAK2 and TYK2 inhibition and is the first oral JAK-i approved for the treatment of RA^[19,20,21]^ and psoriatic arthritis (PsA).^[22]^ Baricitinib is a JAK1 and JAK2 inhibitor used in RA treatment,^[23]^ such as filgotinib^[24,25]^ and upadacitinb,^[26,27]^ that inhibits more selectively JAK1. Given the high number of cytokines signaling through JAK-STAT, the revolutionary impact of oral anti-JAK small molecule therapy lies in being able to simultaneously block multiple cytokines and the related pathways, effectively overcoming the direct block of single cytokines that is at the basis of therapies with biotechnological anti-cytokine drugs (Figure 1). In the context of pSS, this also translates into the possibility of finding and developing effective therapies that target multiple pathways with a single drug, whereas anti-cytokine biotechnological therapies have instead dramatically failed in several clinical trials.^[12]^

The cytokine landscape characterizing pSS is extremely complex and heterogeneous. Among cytokines signaling through JAK-STAT, IL-6, IL-7, IL-21, and IL-23 have been demonstrated to be potentially involved in pSS pathogenesis. The classical pro-inflammatory cytokine IL-6 has been found to be increased in serum, saliva, and tears of patients with pSS and linked to the pathogenesis of the disease.^[28,29,30]^ Despite the pathogenetic contribution of IL-6 in crucial immune processes such as the differentiation and activation of both B and T cells, the use of tocilizumab, a recombinant humanized monoclonal antibody acting as an IL-6 receptor antagonist, did not improve systemic involvement and symptoms over 24 weeks of treatment compared with placebo in a multicenter double-blind randomized placebo-controlled trial in pSS patients.^[31]^ IL-21 is a key cytokine in the type I interferon (IFN) signaling pathway, in the generation of follicular subtypes, and IL-17–producing T helper (Th) cells, as well as in plasma cell differentiation and B-cell activation. High levels of IL-21 have been demonstrated in the pSS sera, which are correlated with lower memory B-cell and higher naïve B-cell percentages.^[32,33]^ RNA sequencing of MSG also demonstrated significantly increased levels of IL-21 and IL-21-inducible genes such as IL-21R, JAK3, STAT1, HLA-B, CCR7, and C-X-C motif ligand 10 (CXCL10) in pSS patients.^[34]^ The increased IL-21 signature gene expression was associated with an increased EULAR Sjögren's Syndrome Disease Activity Index score (ESSDAI)^[35]^ and increased enrichment of B cells, memory B cells, CD4^+^ T cells, and CD8^+^ T cells.^[34]^ Interestingly, the expansion of IL-21 T-follicular-helper (Tfh) under the control of ICOS (inducible co-stimulator) has been demonstrated as a characteristic of pSS with ectopic germinal centers and MALT lymphoma.^[36]^

IL-23A is a pro-inflammatory cytokine required for Th17 and innate lymphoid cells (ILC) 3 maintenance and expansion and the production of type 3 cytokines such as IL-17 and IL-22. Intense IL-23 expression has been demonstrated, by immunohistochemical staining, in submandibular glands of C57BL/6.NOD-Aec1Aec2 mice and in pSS salivary gland biopsies within lymphocytic foci and on epithelial tissues.^[37,38]^ IL-23 increased expression is associated in pSS salivary glands with the increased expression of IL-17 and IL-17 producing cells, mainly Th17 and ILC3.^[37]^ According to the increased IL-23 expression, IL-22 and STAT3 are also significantly increased at both protein and mRNA levels in the inflamed salivary glands of patients with pSS and accompanied by the expansion of IL-22-producing cells.^[37]^ Interestingly, Barone *et al*.^[39]^ by using a virus-induced model of autoantibody formation in the salivary glands of adult mice confirmed the role of IL-22 in pSS pathogenesis, demonstrating that IL-22 provides a mechanistic link between mucosal infection, B-cell recruitment, and humoral autoimmunity. IL-22 receptor engagement was in fact necessary and sufficient to promote differential expression of CXCL12 and CXCL13 in epithelial and fibroblastic stromal cells that, in turn, is pivotal for B-cell recruitment and organization of the TLOs (tertiary lymphoid organs). Accordingly, IL-22 blockade impairs and reverses TLO formation and autoantibody production.

An increasing body of evidence suggests also a significant role of IL-7 axis in pSS, by driving both T-cell responses and B lymphoneogenesis. IL-7 and its receptor IL-7Rα levels have been demonstrated to be increased in pSS salivary glands, the latter correlating with the severity of sialadenitis, and involved in the development of pSS ectopic lymphoid structures.^[40]^ Interestingly, the exogenous administration of IL-7 was able to accelerate pSS onset in a mouse model, whereas pSS development was prevented by blocking IL-7Rα signal, suggesting that therapeutic intervention on this axis, by a direct block or an indirect inhibition of downstream molecules, e.g., JAK-STAT, might be useful in pSS.^[41,42]^ By signaling through its receptor featuring the common IL-7Rα chain, in addition to a specific chain named TSLPR (Thymic stromal lymphopoietin receptor), the pathway of thymic stromal lymphopoietin (TSLP), a novel biomarker for pSS and related lymphoproliferation,^[43,44]^ also involves JAK-STAT, and its effects might be modulated by JAK-i agents. Furthermore, very recent data reported that IL-7 secreted by SGECs under IFN influence may activate T cells in pSS, which in turn secrete IFN-γ, enhancing a vicious circle that amplifies one of the main pathogenetic system in connective tissue diseases, i.e., IFN itself.^[45]^

Among such a rich cytokine networking in pSS, growing evidence supports a prominent role of type I IFN (mainly IFN-α and IFN-β), type II (IFN-γ), and, more recently, type III (IFN-λ) also in pSS pathogenesis, based on studies reporting the upregulation of IFN-regulated genes (IRGs, profiling the so-called IFN signatures) both in peripheral blood and in MSG biopsy specimens.^[46,47,48,49,50]^ IFNs are primarily involved in host defense against infections but also in cell differentiation, proliferation, survival, and death and are crucial regulators of the immune system.^[51]^ The hyperexpression of IFN systems in pSS is sustained by chronic reverberating processes, such as autoantigenic overload and lack of control mechanisms, that act together with genetic susceptibility and epigenetic modifications.^[52]^ Despite the difference between IFNs in signaling via specific cell surface receptor complexes, they activate some common downstream pathways, where JAK-STAT is one of the most crucial ones among them.^[51]^ Targeting JAK-STAT can therefore result in the modulation of biological consequences of IFN activation. In pSS, type I IFN plays a central role in initiating and enhancing inflammation in the context of salivary glands, where it is mainly released by plasmacytoid dendritic cells (pDCs) and SGECs after an exogenous, likely viral or bacterial, or endogenous, e.g., autoantigens, trigger.^[52]^ By the expression of more than 2000 genes, the biological effects of type I IFN are impressive and range from innate responses, such as the maturation of DC with expression of costimulatory molecules, antigen presentation, and upregulation of chemokines, to T and B lymphocyte stimulation and activation.^[51,52]^ Among others, in fact, following type I IFN release, crucial adaptive immune responses occur, e.g., the stimulation of CD8^+^ lymphocytes, the differentiation of Th1 and Th17 lymphocytes, the suppression of T regulatory (Treg) lymphocyte activity, and the induction of B-cell hyperactivity, by both enhancing the B-cell activating factor (BAFF) release and lowering of B-cell receptor (BCR) threshold required for B-cell activation.^[52]^ B-cell dysregulation is one of the most impacting pillars in pSS pathogenesis leading to both autoimmunity and lymphoproliferation that accounts for many clinical manifestations and the risk of lymphoma evolution in about 5%–10% of pSS patients.^[3,4]^ Type II IFN sources in pSS are mainly T lymphocytes, but also NK, B cells, macrophages and, to a less extent, DC, being this system involved in antimicrobial protection, apoptosis, inflammation and tissue damage.^[51]^ Very novel data report the presence of an amplification loop between interleukin IL-7, a pivotal cytokine in T-cell responses and T-cell–dependent activation of SGECs and B lymphocytes in pSS, and IFN-γ, supporting the role of the IFN system as a bridge milestone connecting leading actors in pSS pathogenesis, i.e., SGECs, T and B cells.^[45]^ Recently, many studies also focused on type III IFN, with increasing evidence of possible contribution to both autoimmune and malignant disorders.^[53,54]^

Classically, pSS has been indicated as a type I IFN-driven disease,^[52]^ but discrepancies in different IFN signatures expression between peripheral blood and MSG biopsies have been reported also in the same patient, with a predominant type I IFN signature in the former and type II in the latter samples.^[46]^ Higher IFN-γ transcriptional levels were observed in MSG biopsies of patients developing lymphoma,^[46]^ suggesting a possible contribution of IFN systems also in the prediction of the risk of lymphoma evolution in pSS.^[46,55]^ Other authors reported 3 different subsets of IFN expression in MSG biopsies from pSS patients, i.e., purely type I, purely type II, and a mixed type I/type II pattern and described a link between these different patterns and peculiar clinical manifestations.^[47]^ More recently, a very innovative study^[48]^ proposed a new classification of pSS according to results obtained from a huge multi-omic analysis performed on peripheral blood of pSS patients and healthy volunteers, highlighting a clear centrality of IFN signatures in molecular subsetting of pSS patients. Among 4 different identified clusters, namely C1, C2, C3, and C4, those pSS patients belonging to C1, C3, and C4 were sharply characterized by specific combinations between different magnitudes of type I and type II IFN expression.^[48]^ Although an integration between peripheral blood- and tissue-derived data is mandatory in the next future, considering that all crucial events for pSS development and maintenance occur in salivary glands, this novel approach is the first step for developing precision medicine-driven therapies overcoming heterogeneity issues of pSS that led in the past to the failure of many clinical trials.^[12,48,56]^

With regards to IFN-λ, a higher expression in MSG from pSS, compared with non-pSS sicca control subjects, has been demonstrated,^[49]^ and, more recently, a synergistic effect between IL-29, which belongs to type III IFN system, and IFN-α in the induction of BAFF and CXCL10 by prolonged STAT1 phosphorylation in salivary gland epithelium has been also reported.^[50]^

Data regarding JAK and/or STAT expression in pSS salivary glands are limited. Aota *et al*.^[57]^ demonstrated a strong JAK1 and JAK2 expression, respectively, in ductal and acinar cells of MSG biopsies of pSS patients by immunohistochemical analysis. STAT1^[58,59]^ and STAT3^[37,60,61]^ expression has been found also to be increased in pSS MSG biopsies and linked to IFN-α, IFN-γ, and IL-6 stimulation of the former^[58,59]^ and to IL-22 and IL-17 overexpression^[37,60,61]^ of the latter. Furthermore, very recent data demonstrated that STAT3 is also implicated in epigenetic DNA methylation/hydroxymethylation processes in pSS, mostly affecting IFN-α- and IFN-γ-regulated genes, as well as the oxidative stress pathways, and that JAK-i agents (AG490 and ruxolitinib) were able to reverse the global DNA hydroxymethylation mediated by IFNα, IFNγ, and H_2_O_2_ in human SGECs.^[62]^

## JAK Inhibition in pSS

### Baricitinib

There are no many basic research data about JAK-i in pSS. A recent study^[57]^ demonstrated that baricitinib, a JAK1 and JAK2 inhibitor, suppressed the destruction of acinar cells in the salivary gland of pSS patients by abrogating IFN-γ-induced CXCL10 expression and CXCL10-dependent immune cell infiltration in human salivary gland ductal cells. CXCL10 is a chemokine induced by IFN-γ via JAK-STAT during Th1 immune responses, released by peripheral blood mononuclear cells, fibroblasts, and endothelial cells.^[63]^ CXCL10 and its receptor CXCR3 have been involved in the pathogenesis of pSS since they are up-regulated in pSS MSG and contribute to the chemotaxis of immune cells and their accumulation in the context of inflamed salivary glands.^[64,65,66]^ Beside the strong expression of JAK1 and JAK2 in MSG biopsies of pSS patients,^[57]^ Aota *et al*.^[57]^ demonstrated that baricitinib significantly inhibited IFN-γ-induced CXCL10 production in an immortalized human salivary gland ductal-cell clone and by a western blot analysis showed also its ability to strongly inhibit the phosphorylation of STAT1 and, to a less extent, of STAT3. These data suggested a possible role of JAK-i baricitinib in pSS treatment.

Very recently, the efficacy and safety of baricitinib for active pSS patients have been explored in a pilot non-controlled trial.^[67]^ This study enrolled 11 pSS patients, all fulfilling 2016 ACR (American college of rheumatology)/EULAR (European Alliance of Associations for Rheumatology) classification criteria for pSS, showing a ESSDAI of at least 5. The improvement of ESSDAI of at least 3 points has been considered as the minimal clinical improvement expected. Other measures, such as European pSS Patient Reported Index (ESSPRI), Phisycian Global Assessment Score (PGA), Immunoglobulin G (IgG), and remission/improvement of single-organ manifestations have been also collected and evaluated. Patients were treated with baricitinib 2 mg per day and followed up at 3 months and 6 months after starting the therapy. Baricitinib treatment led to a significant ESSDAI reduction, as did with regard to the ESSPRI and PGA. At 6 months, 88.9% patients achieved minimal clinical improvement of ESSDAI.^[67]^ A decreasing trend in IgG and ESR (Erythrocyte sedimentation rate) levels was also observed. Main clinical manifestations showing improvement compared with baseline were skin rash and arthritis, consistent with the study of baricitinib in active SLE patients,^[68]^ weight loss, anemia, and cytopenia. Two pSS patients with interstitial lung disease (ILD) and cough, short of breath, and dyspnea after exertion were relieved in symptoms, together with an improvement of lung involvement in follow-up HRCT (High-resolution computed tomography) scan. A flare of HBV (hepatitis B virus) was the only adverse event reported.^[67]^ Besides major limitations of the study, mainly the absence of a control group, the treatment with baricitinib appears promising in pSS, and high-quality randomized controlled clinical trials are needed to confirm these preliminary results.

### Filgotinib

Lee *et al*.^[69]^ demonstrated that filgotinib, a selective JAK1 inhibitor, suppressed in pSS the expression of IFN-related genes and of BAFF in human SGECs, as well as the BAFF production in salivary gland organoids. In addition, after filgotinib administration in mice models, both increased salivary flow rates and amelioration of lymphocytic infiltration of salivary glands were reported,^[69]^ suggesting that filgotinib might be a novel therapy to directly target salivary gland inflammation and, possibly, lymphoproliferation in pSS patients.

Results from a multicenter, double-blind placebo-controlled randomized phase 2 clinical trial including an arm with filgotinib, besides other agents (i.e., lanraplenib and tirabrutinib), in order to assess both safety and efficacy in patients with active pSS (ESSDAI ≥ 5), have been very recently published.^[70]^ Patients randomized to the filgotinib arm received the dosage of 200 mg per day for 48 weeks. The primary endpoint was defined as the proportion of patients fulfilling at week 12 both protocol-specified improvement and nonworsening criteria, based on C-reactive protein (CRP) and pSS-related symptoms, assessed by the visual analog scale (VAS) of global disease, pain, oral dryness, ocular dryness, and fatigue. Change in ESSPRI and ESSDAI was included as a secondary endpoint and assessed at week 12 and week 24. Exploratory efficacy endpoints included objective tests, such as Schirmer's test and salivary flow (unstimulated and stimulated), treatment response on specific ESSDAI domains, and ESSDAI score change from baseline in subgroups of patients. Exploratory biomarker-related endpoint was the change from baseline for selected peripheral biomarkers, e.g., IgA, IgG, IgM, rheumatoid factor (RF) and CRP, B cell and plasma cell subset, and IFN signature, for each patient at week 4, 12, and 24.^[70]^

At week 12, 43.3% of the filgotinib group achieved the primary endpoint, although no statistically significant difference was found compared with the placebo arm.^[70]^ Neither secondary endpoint was met. However, some notable evidence emerged from the trial. Change of ESSDAI appeared more pronounced after filgotinib treatment in the pSS subgroup of patients with baseline ESSDAI ≥ 14 or without disease-modifying antirheumatic drugs/corticosteroids. Moreover, by week 24, greater decreases in RF, IgM, IgG, and IgA were seen in the filgotinib group compared with placebo, and, very interestingly, the IFN activity was significantly reduced from baseline at week 4 and week 12. Cytosolic DNA sensing and chemokine signaling pathways were also reduced by filgotinib therapy. In exploratory analyses, the salivary rate and tear production resulted stabilized at similar levels compared with baseline during the treatment with filgotinib.^[70]^ Finally, most adverse events were not severe, and overall safety and tolerability were consistent with the already known safety profile. In light of this, although primary and secondary endpoints were not met, these observations support *post hoc* analyses in specific subgroups of pSS patients, possibly guided by peculiar biomarkers, which, together with a general deep revision of pSS outcome measures for clinical trials, already ongoing,^[71]^ might lead to target more accurately pSS patients and potentially to prove efficacy of promising novel therapeutic agents, such as filgotinib, for use in clinical practice.^[70]^

### Tofacitinib

Autophagy is one additional altered mechanism in pSS, which is involved in several homeostatic functions and both in innate and adaptive immune responses.^[72,73]^ Deficiency of autophagy has been associated with increased inflammation by IL-6 and accumulation of JAK-STAT components.^[74]^ In a recent study,^[75]^ Barrera *et al*.^[75]^ evaluated autophagy dysregulation in pSS and its link with JAK-STAT system by analyzing MSG biopsies from both pSS patients and control subjects and by generating autophagy-deficient (ATG5 knockdown) 3 dimensional (3D) salivary glands acini, which were then incubated in the presence or in absence of the JAK1 and JAK3 blocking agent tofacitinib. MSG from pSS patients showed decreased ATG5 expression, correlating negatively with increased activation of STAT1 and STAT3. Increased expression of STAT1 and IL-6 correlated with ESSDAI and the presence of anti-SSA antibodies.^[75]^ ATG5-deficient 3D-acini showed also increased expression of pro-inflammatory cytokines such as IL-6, interestingly reversed by tofacitinib.^[75]^

### Ruxolitinib

The biological effects of ruxolitinib, a JAK1 and JAK2 inhibitor, on mesenchymal stromal cells (MSCs), isolated from salivary glands of both pSS patients and controls, have been recently evaluated *in vitro*.^[76]^ A ruxolitinib-mediated inhibition of IFN-γ-induced expression of MSC immunomodulatory markers, such as HLA-DR (Major histocompatibility complex (MHC) II cell surface receptor) expression, has emerged by these experimental studies, together with the block of CD4^+^ T cell chemotaxis through the inhibition of MSC production of CXCL9, CXCL10, and CXCL11, suggesting potential implications for ruxolitinib in pSS therapy.^[76]^

## Conclusions

JAK-STAT inhibition has become in recent last years a new therapeutic option approved for clinical use in immune-mediated disorders and tested in an increasing number of clinical trials for other autoimmune diseases, including pSS. The variegate cytokine landscape signaling through the JAKSTAT system and, among others, the prominent importance of IFN pathways in both pSS pathogenesis and patient subsetting suggest the potential role of JAK-i in treating pSS by modulating crucial molecular and biological events for the disease development and maintenance. *In vitro* and *in vivo* data seem to support this hypothesis, together with encouraging results from clinical trials, despite the inadequacy of outcome measures available at the moment. However, many efforts have still to be dedicated to more clearly elucidate the effects of blocking JAK-STAT pathways in pSS, not only in the perspective of controlling inflammation but also in that of rescuing the homeostatic complex functions of the salivary gland epithelium.^[77]^
